# The complete chloroplast genome of a medical herb, *Rheum lhasaense* (Polygonaceae), from Qinghai-Tibet Plateau in China

**DOI:** 10.1080/23802359.2021.2002205

**Published:** 2022-01-18

**Authors:** Ruifeng Zhang, Likuan Liu, Hongyu Wang, Junyan Zhang, Zhenglei Wang, Jinping Li, Yang Zeng

**Affiliations:** a The College of Biological Science, Qinghai Normal University, Xining, China; b Academy of Plateau Science and Sustainability, Xining, China

**Keywords:** *Rheum lhasaense*, chloroplast genome, Polygonaceae, Qinghai-Tibet Plateau, phylogenetic trees

## Abstract

*Rheum lhasaense* (Polygonaceae) is one of the genuine medicinal herbs in Qinghai-Tibet Plateau, China. Here we report the first chloroplast (cp) genome of *R. lhasaense* using Illumina NovaSeq 6000 platform. The length of its complete cp genome is 161,820 bp, containing four sub-regions. A large single copy region (LSC) of 87,086 bp and a small single copy region (SSC) of 12,814 bp are separated by a pair of inverted repeat regions (IRs) of 30,960 bp. The complete cp genome of *R. lhasaense* contains 130 genes, including 85 protein-coding genes, 37 tRNA genes, and 8 rRNA genes. The overall GC content of the cp genome is 37.4%. The phylogenetic analysis, based on 28 cp genomes, suggested that *R. lhasaense* is closely related to *R. acuminatum* and *R. pumilum.*

*Rheum* L. (Polygonaceae) includes about 60 species distributed in the alpine mountains of temperate and subtropical Asia. There are 39 *Rheum* species distributed in China (Li 1998). *Rheum lhasaense* is mainly distributed in the hillsides and meadows area (altitude 4200–4600 m) in central and eastern Tibet. It is one of the common Tibetan medicines. Its dry roots are usually used as medicine to treat constipation, stomachache, hyperlipidemia, cardiovascular, and cerebrovascular disease (Dashang 1997; Lin et al. 2019). The root of *R. lhasaense* didn’t contain anthraquinone, which is a significant difference between *R. lhasaense* and other *Rheum* species (Liu et al. 2013). Despite its importance in medicinal value, there is little genetic information reported for *R. lhasaense.* To study the systematic position and genetic background of *R. lhasaense*, we sequenced *R. lhasaense* DNA and obtained its complete chloroplast (cp) genome.

The voucher specimen of *R. lhasaense* was collected from the hillsides area in the Duodigou, Chengguan District, Lhasa City, Tibet Autonomous Region, China, on August 12 in 2020 (alt. 4231.61 m, E91°11'38“, N29°43'29“), and the specimen was deposited at Herbarium, School of Life Sciences, Zhengzhou University, with a voucher number of ZZU2020-7438. The total DNA was isolated from leaf materials of the specimen using the plant genomic DNA extraction kit (Solarbio LIFE SCIENCES, China). The DNA concentration and quality were then measured by NanoDrop2000c micro-uv spectrophotometer (Thermo Scientific, America). The DNA was sequenced at Novogene Biotech Co. (Beijing, China) using the Illumina NovaSeq 6000 platform with a 150-bp shotgun library. Finally, 1.83 G of 150-bp paired-end raw reads of *R. lhasaense* were obtained, processed and assembled following the method of Nicolas et al. (2017). The raw sequencing reads was deposited in SRA with the no. of PRJNA735018. The assembled contigs were mapped to the reference cp genome (*R. tanguticum*, GenBank accession no. NC_046695) and annotated using Geneious Prime software (https://www.geneious.com). The border regions between the large single copy region (LSC), the small single copy region (SSC) and two inverted repeat regions (IRs) were validated by PCR amplifications and Sanger sequencing, and the results showed that the sequence was correct. The complete cp genome of *R. lhasaense* is 161,820 bp in length, and it released to NCBI (GenBank accession no. MZ328078). It contains two IRs of 30,960 bp, separated by a LSC of 87,086 bp and a small SSC of 12,814 bp. The cp genome of *R. lhasaense* is comprised of 130 genes, including 85 protein-coding genes, 8 rRNA genes, and 37 tRNA genes. The overall GC content of the cp genome is 37.4%, while the corresponding values of the LSC, SSC, and IR regions are 35.5%, 32.7%, and 41.1% respectively.

The cp genome of *R. lhasaense* and 27 cp genome sequences (downloaded from GenBank) were aligned using MAFFT (Katoh and Standley 2013) and were then constructed phylogenetic trees using neighbor-joining (NJ) method in MEGA7 (Kumar et al. 2016). *Ricinus communis* (Euphorbiaceae) and *Euphorbia lathyris* (Euphorbiaceae) were selected as outgroups. The results showed that *R. lhasaense* was closely related to *R. acuminatum* and *R. pumilum*, and *Rheum* were closely related to *Oxyria* (Figure 1). The phylogenetic analysis was consistent with previous studies (Zhou et al. 2020).

**Figure 1.**
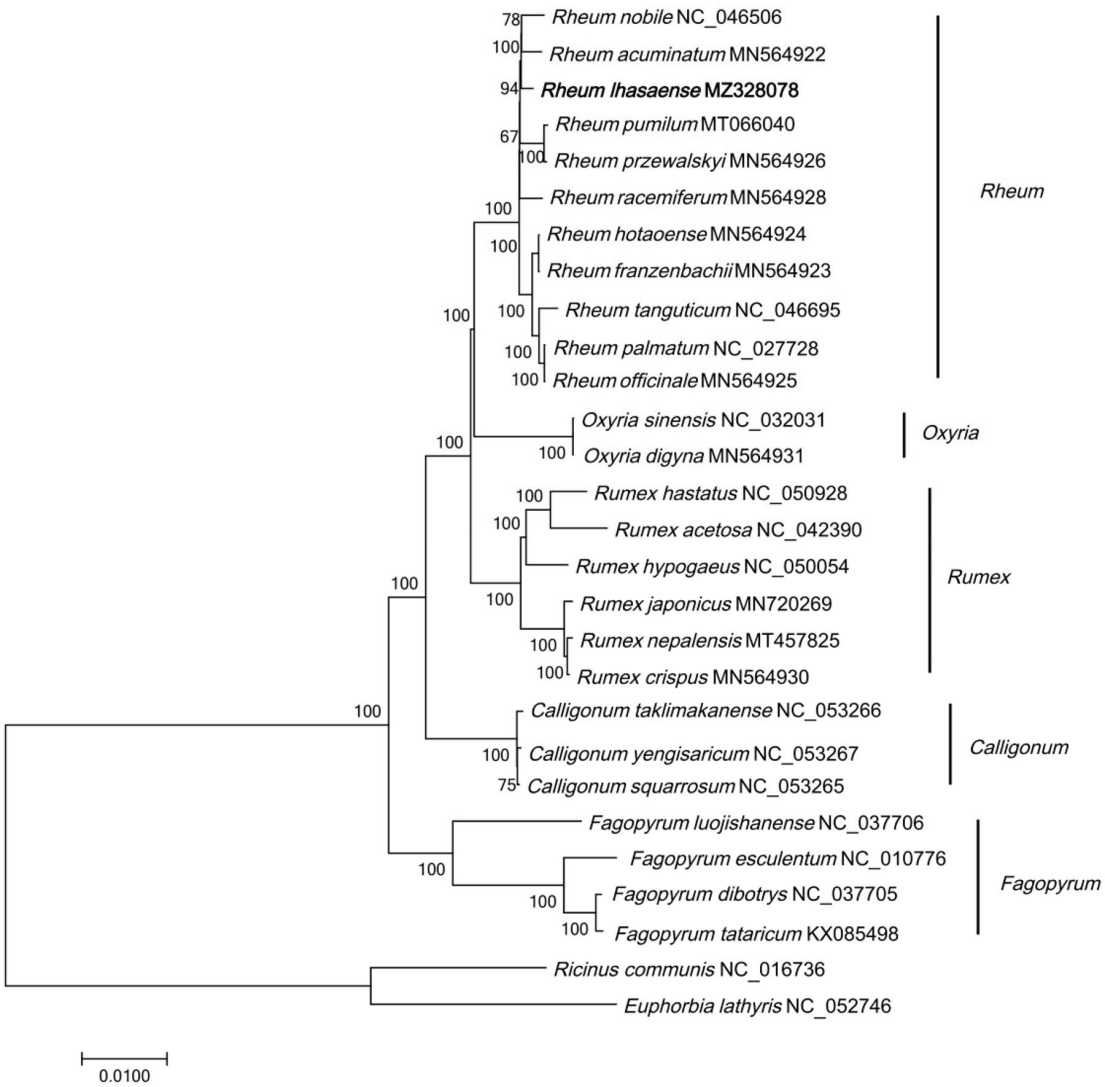
Phylogenetic tree of 28 species based on complete chloroplast genome sequences using NJ (with 1000 replicates) method. The numbers below the branches indicate the corresponding bootstrap support values from the NJ tree. *Ricinus communis* (NC_016736) and *Euphorbia lathyris* (NC_052746) are outgroups.

## Data Availability

The data that support the findings of this study are openly available in GenBank of NCBI at (https://www.ncbi.nlm.nih.gov/), under the accession no. MZ328078. The associated BioProject, SRA, and Bio-Sample numbers are XPRJNA735018, SRA: SRR14727011, and SAMN19553584 respectively.
